# Predicting unfavorable response to initial radioactive iodine therapy in differentiated thyroid cancer: an explainable machine learning and survival analysis approach

**DOI:** 10.3389/fendo.2026.1943597

**Published:** 2026-09-04

**Authors:** Longxuan Gu, Guiwen Zheng, Yuechao Zhao, Jiyuan Wang, Lei Qu, Shuzhan Yao

**Affiliations:** 1 Department of Nuclear Medicine, Shandong Provincial Hospital Affiliated to Shandong First Medical University, Jinan, Shandong, China; 2 Shandong First Medical University, Jinan, China

**Keywords:** differentiated thyroid cancer, machine learning, persistence/recurrence-free survival, radioactive iodine therapy, SHAP

## Abstract

**Background:**

Early post-treatment prognostic assessment may facilitate individualized follow-up in patients with differentiated thyroid cancer (DTC) after initial radioactive iodine (RAI) therapy (RAIT).This study aimed to develop and internally validate a dual-mode machine-learning framework integrating clinical information available from the pre-RAI baseline through the early post-RAI assessment period to predict subsequent unfavorable treatment response and persistence/recurrence-free survival (PRFS).

**Methods:**

A retrospective cohort of 550 DTC patients who underwent total thyroidectomy followed by initial RAIT was included. Multidimensional clinical metrics, including demographic, pathological, imaging, and serum biochemical data, were collected. Post-therapy imaging and biochemical measurements obtained at the first follow-up approximately 1–3 months after RAIT were treated as early post-RAI predictors, whereas subsequent therapeutic response was evaluated dynamically during longitudinal follow-up according to the 2025 American Thyroid Association (ATA) dynamic risk stratification framework. Patients with excellent response (ER) or indeterminate response (IndR) were classified as having a favorable response, whereas those with biochemical incomplete response (BIR) or structural incomplete response (SIR) were classified as having an unfavorable response. PRFS was defined as the interval from initial RAIT to the first subsequent documentation of BIR or SIR; patients without such an event were censored at their last follow-up. Data were randomly split into training and testing cohorts at a 7:3 ratio. Boruta and Least Absolute Shrinkage and Selection Operator (LASSO) were utilized for feature selection in binary classification, while Random Survival Forest (RSF) feature importance ranking was applied for survival modeling. We developed Decision Tree (DT), Random Forest (RF), Light Gradient Boosting Machine (LightGBM), and Multi-Layer Perceptron (MLP)-based Artificial Neural Network (ANN) models for risk classification, alongside the Cox Proportional Hazards (Cox PH) model, Gradient Boosting Survival Analysis (GBSA), RSF, and Extra Survival Trees (EST) for survival prediction. Model performance was evaluated using the Area Under the ROC Curve (AUC), Concordance Index (C-index), calibration curves, and Decision Curve Analysis (DCA). The Shapley Additive exPlanations (SHAP) framework was employed to interpret the models’ decision-making mechanisms.

**Results:**

Among 550 patients, 113 (20.5%) were classified into the unfavorable response group. The ANN classification model demonstrated superior performance on the testing cohort, with an AUC of 0.932 (95% CI: 0.888–0.967), accuracy of 0.879, specificity of 0.947, F1-score of 0.677, and the lowest Brier Score (0.091). Its clinical net benefit, as assessed by DCA, exceeded that of other models. Key predictors in the ANN risk classification model included Extrathyroidal Extension (ETE), T-stage, iodine-avid lymph node status (iodine_LN), RAI Dose, Metastatic Lymph Nodes (MLN), pre-radioiodine therapy stimulated thyroglobulin (pre_sTg), and pre-radioiodine therapy thyroglobulin antibody (pre_TgAb). Regarding PRFS prediction, the RSF model achieved the highest C-index (0.886) and a mean time-dependent AUC of 0.910 (95% CI: 0.867–0.940), outperforming other models. The core prognostic factors for the RSF model included pre_sTg, thyroglobulin at first follow-up (Tg_FU1), Pre-radioiodine Therapy Thyroglobulin/TSH Ratio (Pre_RAI_Tg_TSH_Ratio), MLN, RAI Dose, Thyroid Stimulating Hormone at first follow-up (TSH_FU1), and Thyroglobulin Antibody Reduction Rate (TgAbRR).

**Conclusion:**

In this single-center cohort, the ANN and RSF models demonstrated favorable internal performance for unfavorable-response classification and PRFS prediction, respectively. These findings provide preliminary proof-of-concept evidence for an early post-RAI prognostic assessment framework; however, prospective multicenter external validation is required before its generalizability or clinical utility can be established.

## 1 Introduction

Thyroid cancer (TC) stands as one of the most prevalent malignancies of the endocrine system. In recent years, the incidence of TC has continued to rise (1), driven by the widespread application of advanced imaging modalities, fine-needle aspiration (FNA) biopsy, and continuous improvements in diagnostic technologies. Differentiated thyroid cancer (DTC) remains the most common histological subtype, accounting for over 90% of all cases (2).

Despite its high incidence, studies indicate that TC ranks relatively low in terms of cancer-related mortality in China (3). The estimated number of deaths from TC in China in 2024 was 11,900, with a world-standardized mortality rate of 0.50 per 100,000, which is significantly lower than that of high-burden malignancies such as lung, liver, colorectal, gastric, and esophageal cancers. These data underscore the relatively favorable prognosis of DTC; however, given the large patient population and the long survival duration, the focus of clinical management has shifted beyond early detection and surgical intervention toward comprehensive postoperative follow-up, risk stratification, and long-term endocrine management.

The Department of Nuclear Medicine plays a pivotal role in the holistic management of DTC, providing both precise diagnostic imaging for clinical decision-making and acting as the primary center for postoperative radioactive iodine (RAI) therapy (RAIT). While the majority of DTC patients present with low-grade malignancies and slow disease progression, often achieving a favorable prognosis following total thyroidectomy and initial RAIT, the risk of local recurrence and distant metastasis persists. Approximately 10% to 30% of DTC patients experience recurrence or distant metastasis during long-term follow-up. Notably, while the cancer-specific mortality rate for patients with local recurrence is approximately 8%, it escalates to nearly 50% for those who develop distant metastases (4).

Consequently, TSH-suppressive therapy following RAIT and regular dynamic follow-up are critical components of postoperative management. In clinical practice, appropriate TSH suppression can mitigate the risk of recurrence; however, such interventions must be dynamically tailored based on individual risk stratification, therapeutic response, and the patient’s clinical status. Therefore, addressing the unique clinical profile of DTC, characterized by high incidence, low mortality, and a long management cycle, requires standardizing RAIT and implementing individualized management strategies for the early and precise identification of high-risk patients to maximize long-term clinical benefits.

Currently, the evaluation of RAIT efficacy is often characterized by a temporal lag. Given that the risk of recurrence is dynamic over time, repeated periodic assessments are required to update therapeutic response stratifications. Patients demonstrating a favorable prognosis may benefit from de-escalated interventions and reduced follow-up frequency (5). Furthermore, the insidious nature of micrometastases and the susceptibility of serological markers to immune interference pose significant challenges for traditional experience-based assessment criteria in achieving early, accurate prognosis. Machine Learning (ML) algorithms, with their superior capacity for high-dimensional data mining and capturing complex non-linear associations, offer a promising opportunity to overcome the performance bottlenecks inherent in traditional linear statistical models (6).

In this study, we integrated multidimensional clinical information available from the pre-RAI baseline through the early post-RAI assessment period to develop and internally validate a dual-mode machine-learning framework for early post-RAI prognostic assessment, comprising classification of subsequent unfavorable treatment response and prediction of persistence/recurrence-free survival (PRFS).Our objective was to explore the key risk factors and prognostic determinants in DTC patients following their initial RAIT. Furthermore, we incorporated the SHAP (Shapley Additive exPlanations) framework to demystify the “black box” of algorithmic decision-making. By leveraging these parameters to assess early clinical risk and prognosis, we aim to identify patients with poor outcomes to guide timely clinical interventions, while enabling personalized de-escalation of follow-up frequency and intensity for those with a favorable prognosis, thereby alleviating their financial and psychological burdens.

## 2 Methods

### 2.1 Study population

We retrospectively collected clinical data from 829 patients with DTC who underwent RAIT at the Department of Nuclear Medicine, Shandong Provincial Hospital Affiliated to Shandong First Medical University, between August 2021 and June 2025. Inclusion criteria:(1) Pathologically confirmed DTC following total or near-total thyroidectomy, with or without cervical lymph node dissection; (2) receipt of standardized initial ^131^I therapy; (3) at least 6 months of regular follow-up post-RAI with complete survival time data; and (4) availability of comprehensive imaging and biochemical data to perform the 2025 ATA dynamic risk stratification (7). Exclusion criteria: (1) Strong thyroglobulin antibody (TgAb) positivity (>115 IU/mL, measured by Roche ECLIA) causing severe interference with serum Tg measurement; (2) absence of critical clinicopathological data required for the 8th edition AJCC/UICC TNM staging (8) or 2025 ATA initial risk stratification; (3) presence of other concurrent malignancies or ongoing anti-tumor therapies (e.g., targeted therapy, immunotherapy); and (4) history of external head and neck radiotherapy. Ultimately, 550 patients were enrolled.

This study was conducted in accordance with the Declaration of Helsinki and approved by the Institutional Ethics Committee (Approval SWYX: No.2026-573). Informed consent for RAIT was obtained from all patients. Due to the retrospective nature of the study, the requirement for additional written informed consent was waived by the ethics committee.

### 2.2 Treatment protocol and image acquisition

All patients discontinued levothyroxine (LT4) for at least 4 weeks and adhered to a strict low-iodine diet to ensure serum thyroid-stimulating hormone (TSH) levels reached >30 μIU/mL (Abbott chemiluminescence) prior to RAI. RAI doses were determined by a multidisciplinary team based on individual clinical status, in accordance with the 2015 ATA guidelines and the 2021 Chinese Society of Nuclear Medicine consensus (9) (10). Post-therapy whole-body scintigraphy (I-WBS) and SPECT/CT were performed 3 days after ^131^I administration using a Siemens Symbia Intevo Bold system with a high-energy general-purpose collimator. I-WBS parameters included: anterior/posterior planar imaging, scan speed 15 cm/min, 256×256 matrix, 364 keV energy peak, and 20% window. SPECT/CT involved automatic contour-based tomography (15 s/frame, 64×64 matrix) and a helical CT scan (5 mm slice thickness, 300 mA, 120 kV). TSH-suppressive therapy was initiated within 24–72 hours post-RAI.

### 2.3 Data collection and variable definitions

Data were extracted from electronic medical records and laboratory information systems. Predictor variables were categorized as follows:

#### 2.3.1 Demographics and metabolic metrics

Gender, age, body mass index (BMI), and comorbidities (hypertension, diabetes, Hashimoto’s thyroiditis). Metabolic factors, including fasting plasma glucose, triglycerides, cholesterol, and HDL/LDL, were recorded to calculate the Triglyceride-Glucose (TyG) index.

#### 2.3.2 Imaging assessment

Postoperative neck ultrasound/CT, post-RAI I-WBS, and/or SPECT/CT were used to evaluate remnant thyroid tissue and the location, size, and iodine avidity of metastatic lesions. Images were independently reviewed by two experienced radiologists blinded to clinical outcomes; discrepancies were resolved by consensus.

#### 2.3.3 Clinicopathological and serological dynamics

Pathological subtypes, tumor diameter, multifocality, bilateral involvement, and extrathyroidal extension (ETE) were recorded. TNM staging followed the 8th edition system. Lymph node status, including metastatic lymph node (MLN) count, total dissected nodes, and lymph node ratio (LNR), was documented, alongside the presence of iodine-avid lymph nodes (iodine_LN) on I-WBS. Patients were stratified into four categories based on the 2025 ATA guidelines: Low, Low-Intermediate, Intermediate-High, and High risk. Therapeutic parameters, including initial RAI dose and time interval to initial RAI, were also included. For serological dynamics, two time points were established: (1) Pre-RAI Baseline: 1 day before RAI (TSH >30 μIU/mL) to measure pre-radioiodine therapy stimulated thyroglobulin (pre_sTg), pre_TgAb, and the Pre_RAI_Tg_TSH_Ratio; (2) First Post-therapy Assessment: 1–3 months post-RAI (under LT4 suppression), recording the interval (RAI_FU1_interval), Tg_FU1, TSH_FU1, and TgAb_FU1. Dynamic changes were calculated as follows:


$$TgRR=\frac{sTg-Tg\_\text{FU}1}{sTg}\times 100\%$$

$$TgAbRR=\frac{\text{pre}\_TgA\text{b}-\text{TgAb}\_\text{FU}1}{\text{pre}\_TgA\text{b}}\times 100\%$$

Post-therapy imaging and the first biochemical follow-up constituted the early post-RAI predictor assessment period; all first follow-up assessments were completed within approximately 3 months after RAIT.

### 2.4 Outcome assessment

Therapeutic response was evaluated longitudinally after the early post-RAI assessment period according to the 2025 ATA Dynamic Risk Stratification (DRS) framework and categorized as: (1) excellent response (ER); (2) indeterminate response (IndR); (3) biochemical incomplete response (BIR); or (4) structural incomplete response (SIR). The first biochemical assessment at approximately 1–3 months after RAIT was considered an early post-treatment assessment rather than the definitive response-to-therapy evaluation, as patients were still undergoing initial levothyroxine dose adjustment and TSH-suppressive management during this period. Patients with ER or IndR were classified as having a favorable response, whereas those with BIR or SIR were classified as having an unfavorable response. Persistence/recurrence-free survival (PRFS) was defined as the interval from initial RAIT to the first subsequent documentation of BIR or SIR during longitudinal follow-up; patients without such an event were censored at the date of their last follow-up.

### 2.5 Data preprocessing

The full dataset was randomly partitioned into a training cohort and a testing cohort at a 7:3 ratio using stratified sampling based on the patient’s therapeutic response. The training cohort was exclusively used for feature selection, model development, and hyperparameter optimization, whereas the testing cohort was reserved for internal evaluation of model performance. Given the distinct modeling objectives and outcome formats for early risk classification versus prognostic survival prediction, independent feature selection procedures were implemented to tailor predictor selection to the objective and outcome structure of each modeling task.

#### 2.5.1 Feature selection for risk classification

To identify key predictors of BIR/SIR following initial RAIT, we integrated the Boruta algorithm with Least Absolute Shrinkage and Selection Operator (LASSO) regression (11, 12). First, the Boruta algorithm (500 iterations; P< 0.005) was applied to compare the Z-scores of real features against shadow features, excluding marginal variables to retain only those with robust contributions. Subsequently, LASSO regression was performed using 10-fold cross-validation (CV), with the optimal regularization parameter (λ) selected via the one-standard-error (1-SE) rule to extract non-zero coefficient features. The intersection of features identified by both methods was designated as the final predictor set.

#### 2.5.2 Feature selection for survival prognosis

For PRFS prediction, we employed the permutation importance algorithm based on the Random Survival Forest (RSF) (13). Initial baseline models were fitted using the full feature set. Variable contribution was quantified by measuring the degradation in model performance following repeated random permutations of individual feature distributions. Features were then ranked by importance, and a forward selection strategy was applied. To prevent data leakage and ensure objective assessment, performance was evaluated dynamically within the training cohort using a 5-fold CV. We monitored the trajectory of the CV C-index as features were added, selecting the feature subset corresponding to the first significant local maximum that approached the global optimum, thereby balancing predictive power with parsimonious generalizability.

### 2.6 Machine learning algorithms

Models were trained and optimized on the training cohort, with performance validated on the independent testing cohort. For the risk classification task, we developed Decision Tree (DT), Random Forest (RF), LightGBM, and Artificial Neural Network (ANN) models, with the ANN implemented as a feedforward Multi-Layer Perceptron (MLP) classifier (14). For survival prognosis, we constructed Cox Proportional Hazards (Cox PH), Gradient Boosting Survival Analysis (GBSA), RSF, and Extra Survival Trees (EST) models (15). Hyperparameters were optimized via grid search combined with 5-fold stratified CV, aiming to maximize the area under the ROC curve (AUC) for classification and the C-index for survival prediction.

Model performance was assessed through a multidimensional strategy. For classification, we utilized the ROC curve, calibration curve, and Decision Curve Analysis (DCA) on the testing cohort to evaluate discriminative ability, calibration, and clinical net benefit. Further metrics included accuracy, sensitivity, specificity, F1-score, positive predictive value (PPV), negative predictive value (NPV), and the Brier score. Detailed definitions, mathematical formulations, and clinical interpretations of these evaluation metrics are provided in the **Supplementary Methods**.

For survival models, we calculated the global C-index for both the training cohort and the testing cohort. We mapped the trajectories of time-dependent AUC across the follow-up period, reporting mean AUC with 95% confidence intervals derived from Bootstrap resampling. Dynamic predictive capability was further assessed at clinical milestones (9, 12, 18, and 24 months) using time-dependent ROC curves, calibration curves, and DCA. Time-dependent DCA and calibration plots incorporated Kaplan–Meier estimates to account for censoring bias, thereby providing a more rigorous assessment of net clinical benefit across varying threshold probabilities. The final selection of optimal models was based on a holistic evaluation of discrimination, calibration, clinical utility, stability, and computational efficiency.

### 2.7 SHAP interpretability analysis

To demystify the “black box” nature of machine learning, the SHAP framework was utilized to quantify feature contributions at both global and individual levels (16). Feature importance, decision plots, and summary beeswarm plots were generated to visualize the magnitude, direction, and distribution of feature effects. Dependence plots were employed to uncover potential non-linear associations and threshold effects between critical variables and outcomes. Furthermore, SHAP waterfall plots were constructed to illustrate the individual-level decision-making process, demonstrating how specific clinical features shift the predicted risk from baseline, thereby enhancing the transparency and clinical interpretability of the model.

### 2.8 Statistical analysis

Data analysis was performed using Python 3.10.4 and R 4.5.1. Continuous variables were expressed as mean ± standard deviation (SD), or median with interquartile range (IQR), and comparisons between groups were performed using Student’s t-test or the Mann–Whitney U test, as appropriate. Categorical variables were presented as frequencies and percentages and were compared using the chi-square (χ²) test or Fisher’s exact test. All statistical tests were two-tailed, and a P value< 0.05 was considered statistically significant.

## 3 Results

### 3.1 Patient characteristics

This study retrospectively enrolled 550 patients with DTC who underwent initial RAIT, of whom 113 (20.5%) were categorized into the unfavorable response group (BIR/SIR). Baseline characteristics were compared between the training cohort and testing cohort (**Supplementary Table 1**). All first post-RAI follow-up assessments were completed within approximately 3 months after RAIT, whereas BIR/SIR events were documented starting from 6 months after iodine therapy. The median follow-up duration, estimated using the reverse Kaplan–Meier method, was 18.46 months (95% CI, 16.82–20.30 months). The distributions of most demographic, laboratory, and clinicopathological variables were consistent between the two cohorts (all P > 0.05), with significant differences observed only in T-stage and initial ATA risk stratification, confirming the balanced and comparable nature of the randomized partitioning. The overall study population had a median age of 42.0 years (IQR: 34.0–51.75 years) and a mean BMI of 25.34 ± 3.92 kg/m², with 175 (31.82%) males. After stratified random splitting, 385 patients were assigned to the training cohort and 165 to the testing cohort.

**Figure 1:**
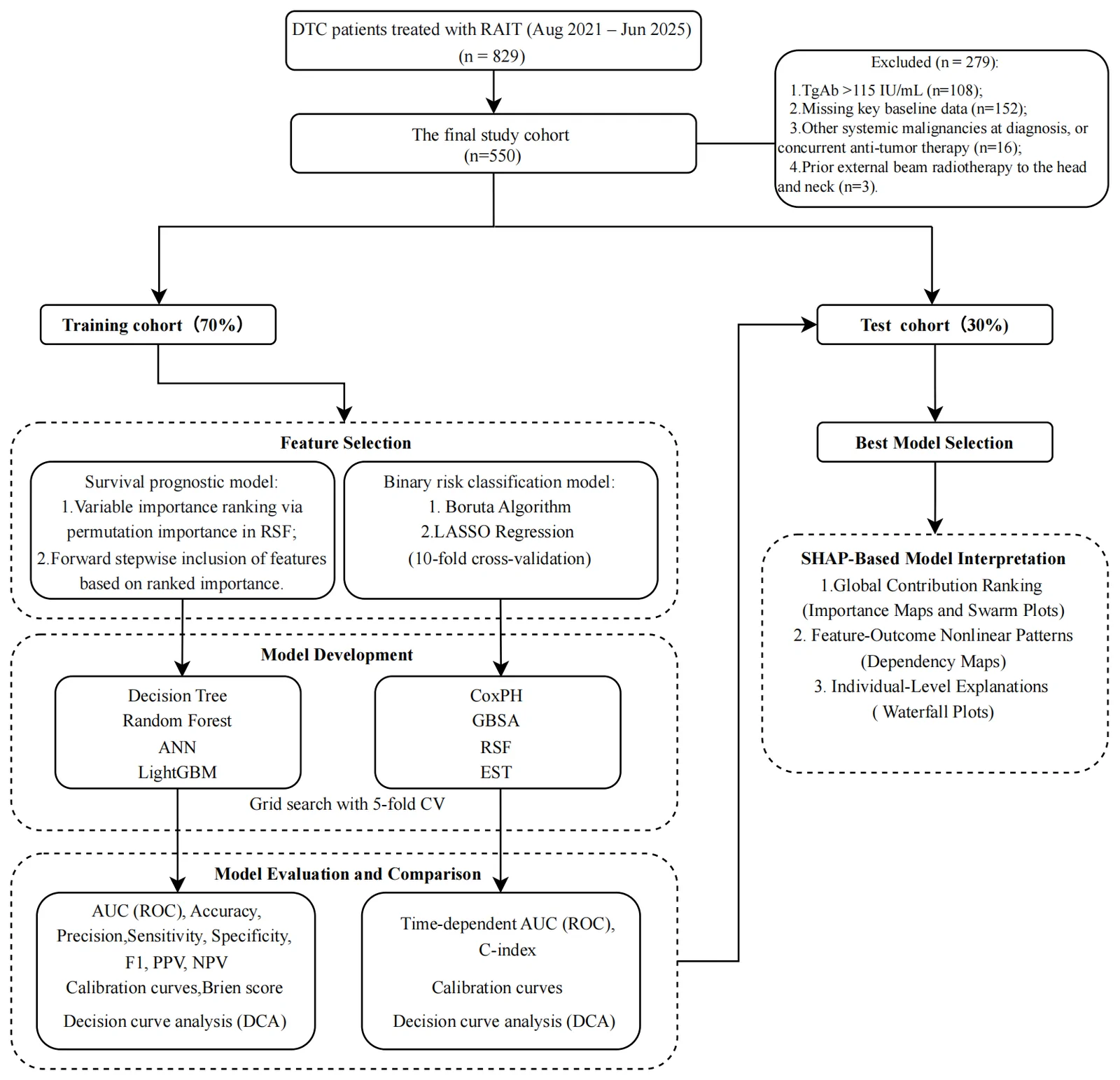
Flow chart of the study design.

In the training cohort (**Supplementary Table 2**), patients in the unfavorable response group exhibited significant differences in multiple baseline characteristics compared to the favorable response group (all P< 0.05). Unfavorable response patients presented with significantly higher LDL and HDL levels, larger tumor diameters, a higher number of MLN, higher total examined lymph nodes (ELN), and an increased lymph node ratio (LNR). Furthermore, pre-RAI sTg, Pre_RAI_Tg_TSH_Ratio, and Tg_FU1 were significantly elevated in the unfavorable response group, whereas pre-RAI and follow-up TgAb levels were relatively lower. Additionally, this group experienced significantly shorter PRFS. Significant differences (all P< 0.05) were also observed in the prevalence of Hashimoto’s thyroiditis, RAI dose, presence of ETE, TNM stage, AJCC stage, iodine-avid lymph node status, and initial ATA risk stratification. The study flowchart is presented in **Figure 1**.

### 3.2 Performance evaluation and selection of ML models for risk classification

**Figure 2:**
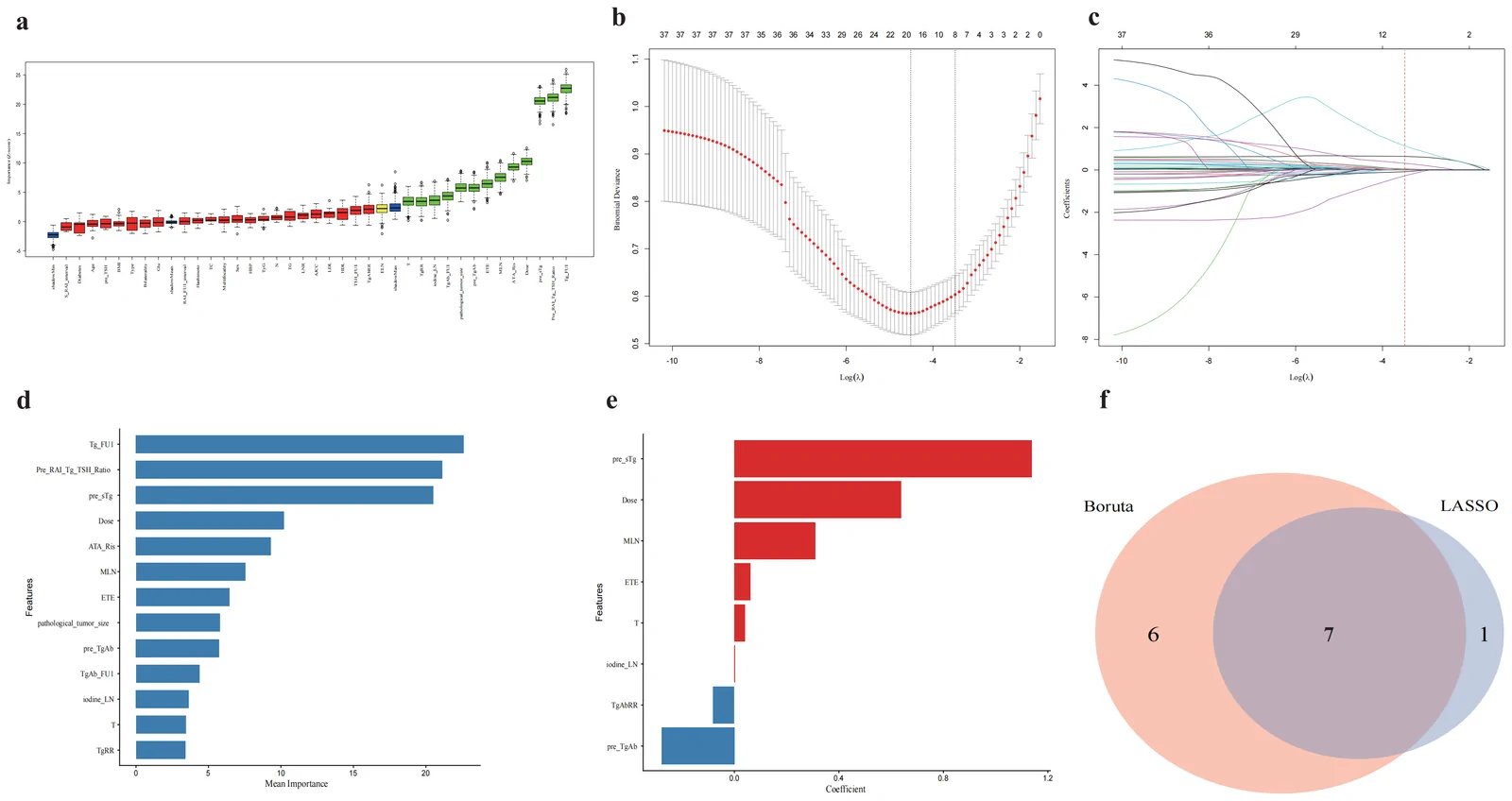
Feature selection and variable identification for the risk classification model. **(a)** The Boruta algorithm was employed to rank the importance of potential clinical features based on Z-scores; features with Z-scores higher than the shadow features were considered significant. **(b)** 10-fold cross-validation of the LASSO regression model, where the vertical dashed line on the right indicates the optimal regularization parameter (λ) chosen according to the one-standard-error rule. **(c)** The shrinkage process of coefficients in the LASSO regression model as the penalty parameter (λ) increases; the vertical red dashed line indicates the optimal log(λ) value. **(d)** The importance ranking of variables based on the Boruta algorithm. **(e)** The coefficients of the 7 selected predictors derived from the LASSO model. **(f)** A Venn diagram illustrating the intersection of features identified by the Boruta algorithm and LASSO regression, resulting in 7 final variables for the risk classification model.

As illustrated in **Figure 2**, feature selection was performed by integrating the Boruta algorithm and LASSO regression. The Boruta algorithm identified 13 significant features, while 10-fold cross-validation of the LASSO regression (based on the 1-SE rule; optimal λ = 0.03059) yielded 8 features with non-zero coefficients. By taking the intersection of these two sets, we identified 7 final predictors: ETE, T-stage, iodine_LN, Dose, MLN, pre_sTg, and pre_TgAb.

**Figure 3:**
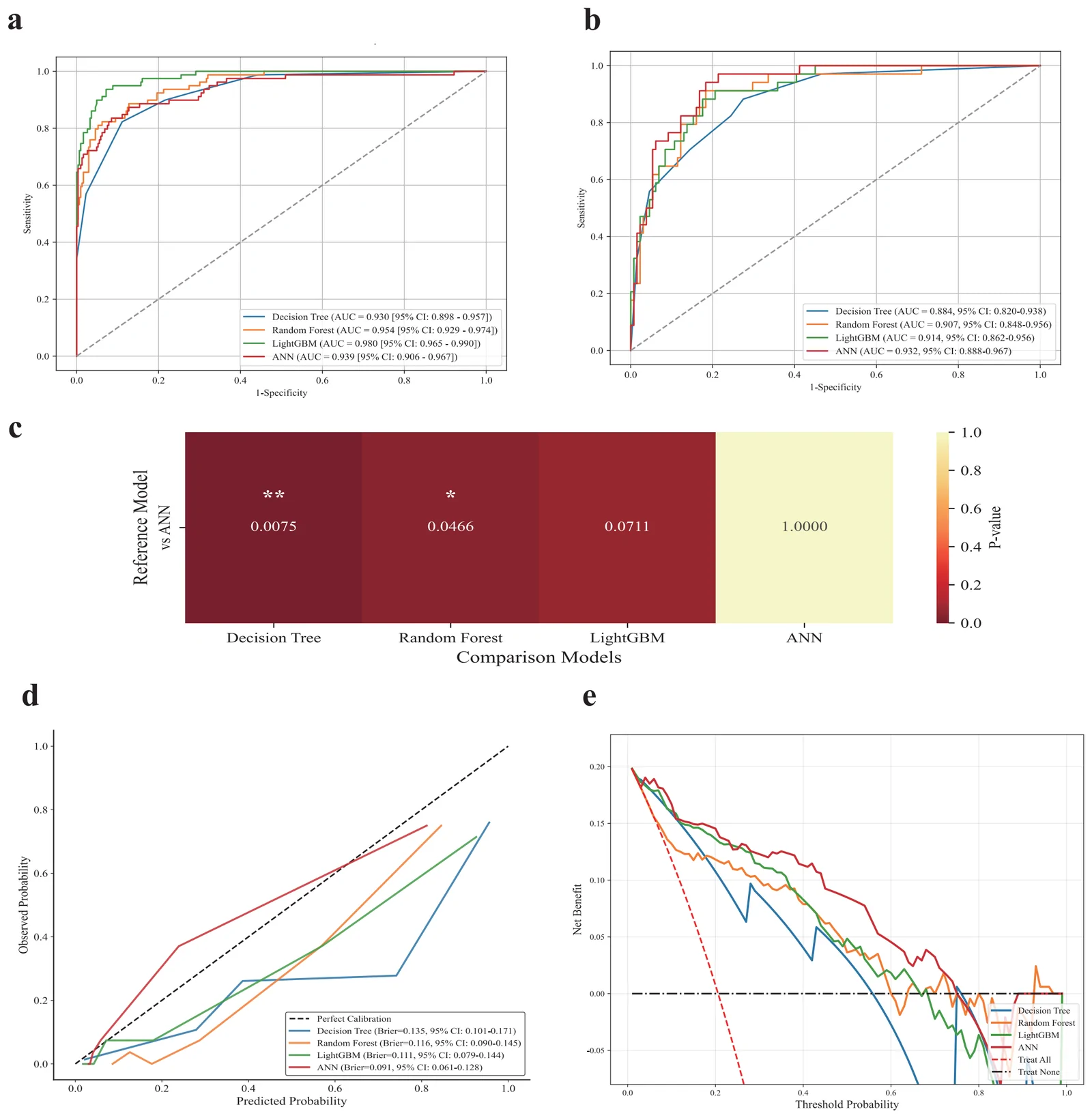
Performance evaluation and comparison of machine learning models for early risk classification. **(a)** Receiver Operating Characteristic (ROC) curves of four classification models in the training cohort. **(b)** ROC curves and Area Under the Curve (AUC) values of the models in the testing cohort. **(c)** DeLong test heatmap comparing the discriminative performance of the ANN model against other models; asterisks denote statistical significance (**P< 0.01, *P< 0.05). **(d)** Calibration curves of the models in the testing cohort, with Brier scores indicating the accuracy of probability prediction. **(e)** Decision Curve Analysis (DCA) compares the clinical net benefit of different models across varying threshold probabilities.

Four ML classification models (DT, RF, LightGBM, and ANN) were developed using the training cohort and validated in the testing cohort. The ANN was implemented as a feedforward Multi-Layer Perceptron (MLP) classifier, and the optimized architecture comprised two hidden layers with 10 neurons each. Given the class imbalance in the cohort, with unfavorable responses accounting for 20.5% of patients, algorithm-specific class weighting was incorporated where supported. DT and LightGBM used balanced class weighting, RF used balanced_subsample weighting, whereas the MLP classifier was trained without class weighting. Detailed hyperparameter search spaces, final configurations, and class-weight settings for all classification models are provided in **Supplementary Table 3**. The ROC curves for the training cohort and the testing cohort are presented in **Figures 3a, b**, respectively.

In the testing cohort, the ANN model demonstrated superior discriminative ability, achieving the highest AUC of 0.932 (95% CI: 0.888–0.967). Regarding comprehensive performance metrics (**Table 1**), the ANN model consistently outperformed the other models, yielding the highest accuracy (0.879; 95% CI: 0.824–0.927), specificity (0.947; 95% CI: 0.904–0.984), F1-score (0.677; 95% CI: 0.531–0.800), and Kappa (0.604; 95% CI: 0.439–0.753). Although LightGBM and RF exhibited higher sensitivity, their relatively lower specificity and PPV reflected a greater proportion of false-positive classifications; in contrast, the ANN model exhibited higher specificity and PPV at the prespecified 0.50 threshold. At the same threshold, the ANN achieved an NPV of 0.905, indicating that 90.5% of patients classified as having a favorable response actually had a favorable outcome in the testing cohort. This provides clinically relevant information on the reliability of favorable-response predictions. To further validate differences in discriminatory performance, DeLong tests were conducted using the ANN model as the reference (**Figure 3c**). The heatmap confirmed that the ANN model significantly outperformed both DT (P = 0.0075) and RF (P = 0.0466). While the AUC difference between ANN and LightGBM did not reach statistical significance (P = 0.0711), the ANN model demonstrated a clear advantage in overall classification performance.

**Table 1:** Detailed performance metrics of various machine learning models.

Dataset category	Model	AUC (95% CI)	Accuracy	Sensitivity	Specificity	F1 score	Kappa	Youden’s J index	PPV	NPV
Training cohort										
	Decision Tree	0.930 (0.898-0.957)	0.875 (0.844-0.909)	0.823 (0.733-0.905)	0.889 (0.851-0.923)	0.730 (0.654-0.798)	0.651 (0.563-0.736)	0.712	0.657	0.951
	Random Forest	0.954 (0.929-0.974)	0.878 (0.844-0.909)	0.835 (0.750-0.917)	0.889 (0.851-0.923)	0.737 (0.662-0.809)	0.659 (0.569-0.749)	0.724	0.660	0.954
	LightGBM	0.980 (0.965-0.990)	0.774 (0.730-0.816)	0.987 (0.956-1.000)	0.719 (0.668-0.768)	0.642 (0.565-0.709)	0.505 (0.425-0.585)	0.706	0.476	0.995
	ANN	0.939 (0.906-0.967)	0.922 (0.894-0.946)	0.722 (0.620-0.810)	0.974 (0.955-0.990)	0.792 (0.710-0.857)	0.744 (0.647-0.822)	0.695	0.877	0.931
Test cohort										
	Decision Tree	0.884 (0.820-0.938)	0.824 (0.770-0.879)	0.706 (0.545-0.864)	0.855 (0.792-0.914)	0.623 (0.486-0.733)	0.511 (0.354-0.651)	0.561	0.558	0.918
	Random Forest	0.907 (0.848-0.956)	0.776 (0.709-0.842)	0.912 (0.800-1.000)	0.740 (0.662-0.817)	0.626 (0.504-0.732)	0.488 (0.351-0.618)	0.652	0.477	0.970
	LightGBM	0.914 (0.862-0.956)	0.794 (0.727-0.855)	0.912 (0.800-1.000)	0.763 (0.687-0.833)	0.646 (0.521-0.752)	0.517 (0.377-0.642)	0.675	0.500	0.971
	ANN	0.932 (0.888-0.967)	0.879 (0.824-0.927)	0.618 (0.448-0.778)	0.947 (0.904-0.984)	0.677 (0.531-0.800)	0.604 (0.439-0.753)	0.564	0.750	0.905

AUC, area under the receiver operating characteristic curve; CI, confidence interval; DT, Decision Tree; RF, Random Forest; LightGBM, Light Gradient Boosting Machine; ANN, Artificial Neural Network, implemented as a feedforward multi-layer perceptron (MLP); PPV, positive predictive value; NPV, negative predictive value; κ, Cohen’s kappa coefficient. Youden’s J index was calculated as sensitivity + specificity − 1. All threshold-dependent metrics were calculated using a fixed predicted-probability cutoff of 0.50 (≥0.50, unfavorable response;<0.50, favorable response).

**Figure 3d** displays the calibration curves and Brier scores for the testing cohort. The ANN model achieved the lowest Brier score (0.091; 95% CI: 0.061–0.128), indicating the smallest mean squared error between predicted and actual BIR/SIR probabilities, thus providing the most precise probability estimation. Furthermore, DCA (**Figure 3e**) revealed that the ANN model consistently yielded higher net clinical benefit than the “Treat All” strategy, “Treat None” strategy, and the other three ML models across a wide range of risk threshold probabilities (approximately 10% to 80%).

Considering the integrated evaluation of AUC, F1-score, DeLong test results, Brier score, and DCA net benefit, the ANN model demonstrated optimal discrimination, calibration, and clinical utility. Consequently, the ANN model was selected as the optimal risk classification tool for predicting early suboptimal response in patients with DTC following initial RAIT.

### 3.3 Performance evaluation and selection of ML models for survival prognosis

**Figure 4:**
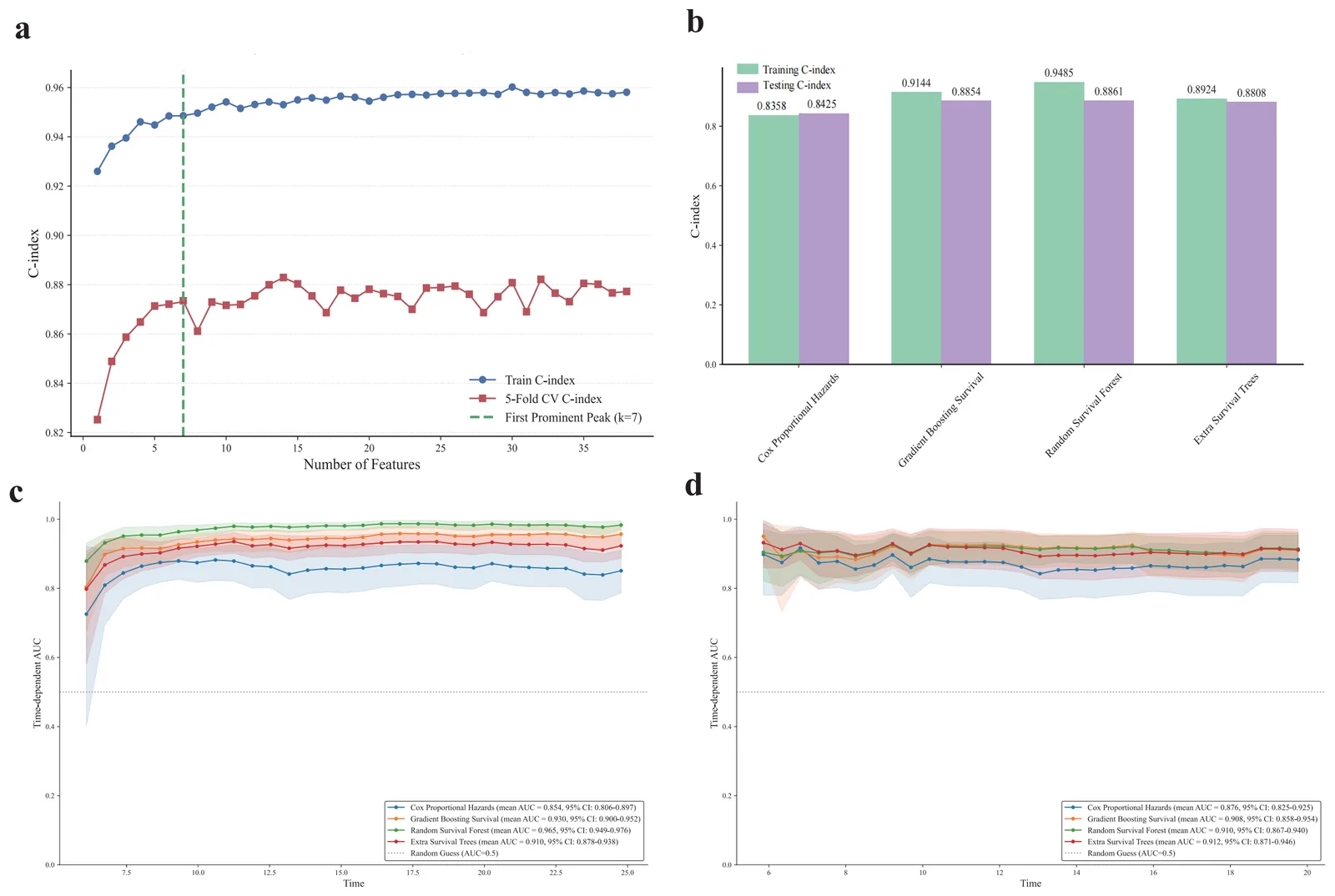
Development and performance comparison of machine learning models for persistence/recurrence-free survival (PRFS) prediction. **(a)** Feature selection process using the Random Survival Forest (RSF)-based permutation importance algorithm. The optimal feature subset (k=7) was determined at the first significant local maximum of the 5-fold cross-validation (CV) C-index before its decline. **(b)** Comparison of the C-index across four survival models in both the training and testing cohorts. **(c)** Time-dependent AUC trajectories of the survival models in the training cohort, including 95% confidence intervals derived from Bootstrap resampling. **(d)** Time-dependent AUC trajectories of the models in the testing cohort, demonstrating the discriminative stability of RSF and other models over time.

To predict PRFS in patients who developed early biochemical or structural incomplete response (BIR/SIR), we employed a variable selection strategy integrating the Random Survival Forest (RSF)-based permutation importance algorithm with a forward selection approach. As shown in **Figure 4a**, although the C-index in the training cohort increased with the number of features, we monitored the performance via 5-fold cross-validation (CV) within the training cohort to rigorously prevent overfitting. The CV C-index reached its first significant peak (0.875) upon the inclusion of seven features, followed by a substantial decline. Further inclusion of features failed to improve generalization performance and suggested an onset of overfitting. Consequently, the top seven features (pre_sTg, Tg_FU1, Pre_RAI_Tg_TSH_Ratio, MLN, Dose, TSH_FU1, and TgAbRR) were locked for subsequent survival analysis.

Based on these seven predictors, four ML survival models (Cox PH, GBSA, RSF, and EST) were constructed using the training cohort. **Figure 4b** illustrates the overall C-index across both the training cohort and the testing cohort. In the testing cohort, the RSF model demonstrated superior survival discrimination (C-index = 0.8861), exhibiting slightly better discriminative performance than the other models.

**Figure 5:**
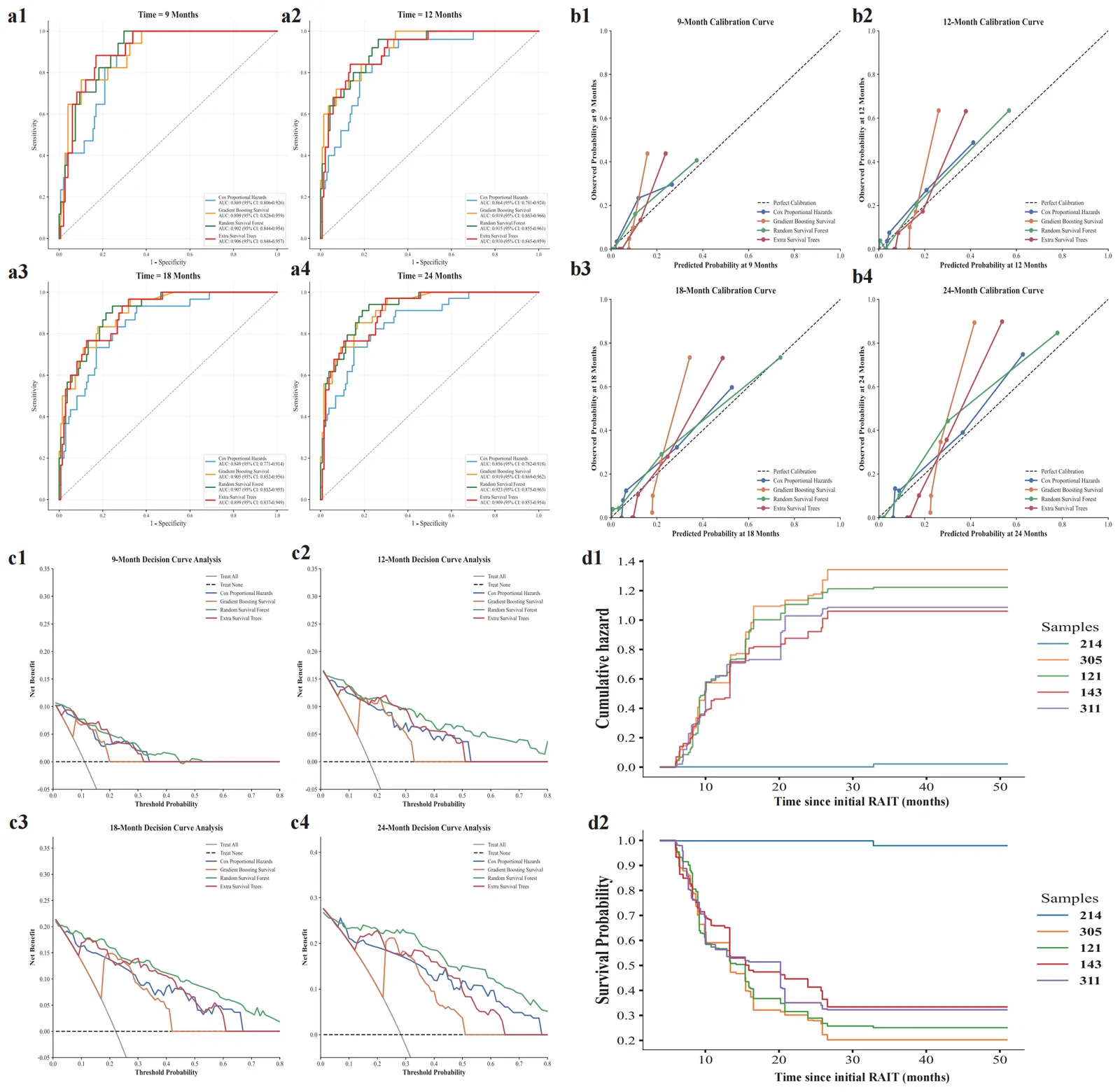
Dynamic prognostic assessment and individualized survival analysis of machine learning survival models. **(a1–a4)** Time-dependent Receiver Operating Characteristic (ROC) curves evaluated at 9, 12, 18, and 24 months, showing the discriminative performance of the models at specific clinical follow-up milestones. **(b1–b4)** Time-dependent calibration curves at the corresponding milestones, comparing predicted versus observed event probabilities. **(c1–c4)** Time-dependent Decision Curve Analysis (DCA) assessing the clinical net benefit of the models at 9, 12, 18, and 24 months. **(d1–d2)** Visualization of individualized **(d1)** cumulative hazard and **(d2)** survival probability trajectories for five randomly selected patients from the testing cohort, demonstrating the model’s ability to capture prognostic heterogeneity.

To further evaluate the dynamic evolution of predictive discrimination, we plotted the time-dependent AUC trajectories for both the training cohort and the testing cohort (**Figures 4c, d**). The results indicated that the RSF model consistently maintained a leading advantage across most follow-up intervals in the testing cohort, with a mean AUC of 0.910 (95% CI: 0.867–0.940). Furthermore, cross-sectional analysis at clinical follow-up milestones (9, 12, 18, and 24 months; **Figures 5a1–a4**) revealed that the RSF model consistently achieved high-tier AUC values. Time-dependent calibration curves (**Figures 5b1–b4**) demonstrated that the predicted risks from the RSF model aligned most closely with actual observed risks compared to traditional Cox models, whereas GBSA and EST exhibited notable deviations at certain time points. Time-dependent DCA (**Figures 5c1–c4**) further confirmed that utilizing the RSF model to guide clinical intervention planning yielded greater net clinical benefit across a wide range of threshold probabilities at each time node.

Given its performance in terms of the C-index, time-dependent AUC, calibration, and DCA, the RSF model was selected as the final model for PRFS prediction following initial RAIT. To illustrate individualized RSF predictions, we randomly selected five patients from the testing cohort and plotted their individualized cumulative hazard (**Figure 5d1**) and survival probability (**Figure 5d2**) trajectories over time. The corresponding predictor values, observed PRFS event status, and event o censoring times for these five patients are provided in **Supplementary Table 4**. These examples illustrate the heterogeneity of model-predicted prognostic trajectories among individual patients and provide a visual demonstration of the model output.

### 3.4 SHAP-based interpretation of the ANN and RSF models

**Figure 6:**
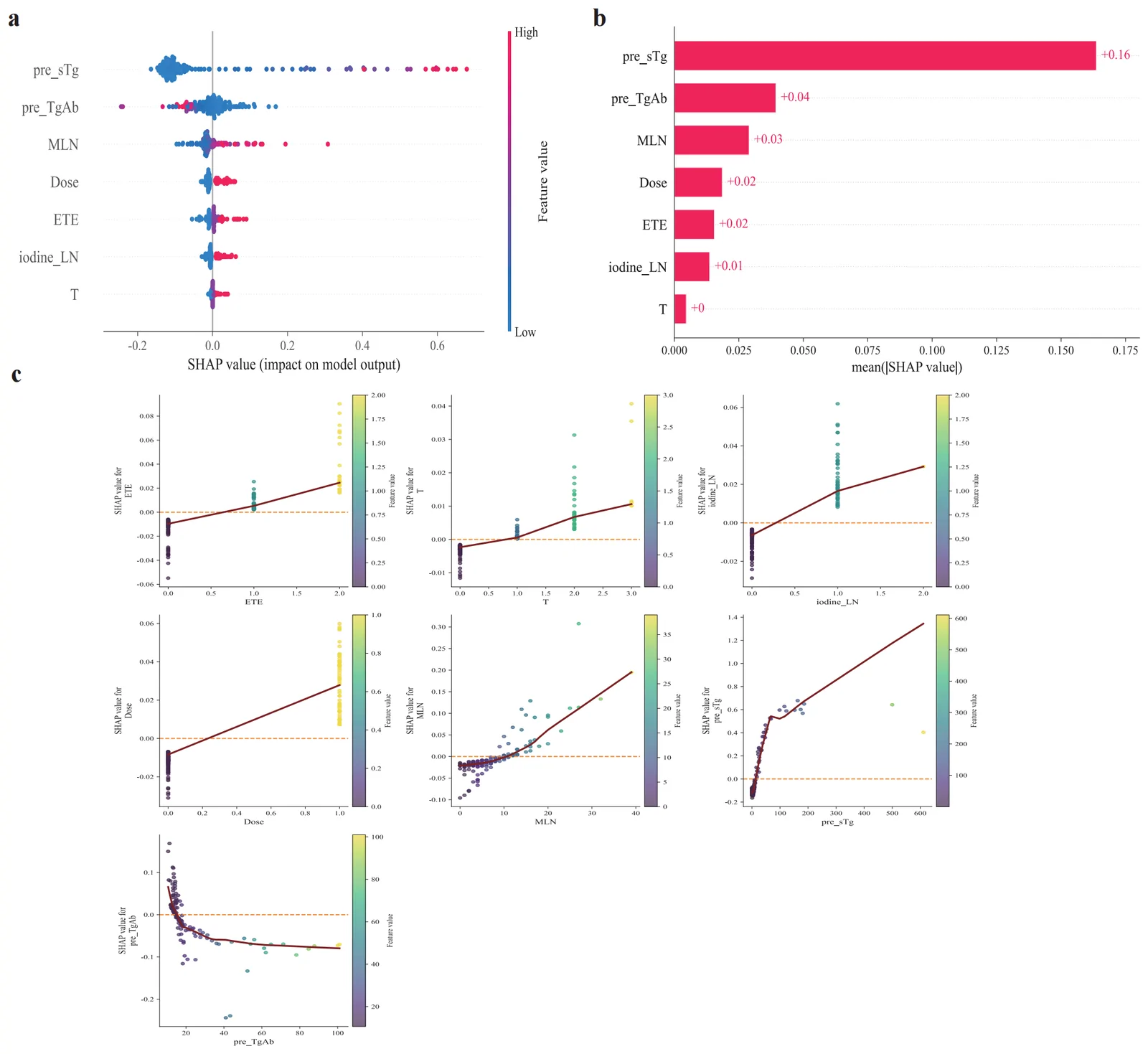
SHAP-based interpretability analysis of the ANN risk classification model. **(a)** SHAP beeswarm plot illustrating the distribution of the impact of each feature on the model output. Each point represents an individual patient; the color indicates the feature value (blue for low, red for high), and the horizontal position shows the impact on the predicted risk. **(b)** Global feature importance measured by the mean absolute SHAP values, quantifying the contribution of each variable to the model’s prediction. **(c)** SHAP dependence plots revealing the non-linear relationship between key clinical features and the model output (SHAP value).

SHAP analysis was used to evaluate the contribution of individual predictors to model output at both the global and patient-specific levels. For the ANN classification model, positive SHAP values indicated that a feature shifted the prediction toward an unfavorable treatment response (BIR/SIR), whereas negative values shifted the prediction toward a favorable response. As shown in **Figures 6a, b**, pre_sTg, MLN, and RAI dose made relatively prominent contributions to the ANN predictions, while pre_TgAb, ETE, iodine_LN, and T-stage also contributed to model classification. Higher pre_sTg levels and greater numbers of MLN were generally associated with a higher predicted probability of BIR/SIR. The dependence plot showed a non-linear relationship between MLN and model output, with SHAP values increasing more noticeably when the number of metastatic lymph nodes approached approximately 10 (**Figure 6c**). In contrast, pre_TgAb generally showed an opposite contribution pattern. These findings suggest that the ANN classification was mainly informed by pre-RAI biochemical tumor burden, nodal disease extent, and other pathological and treatment-related factors.

**Figure 7:**
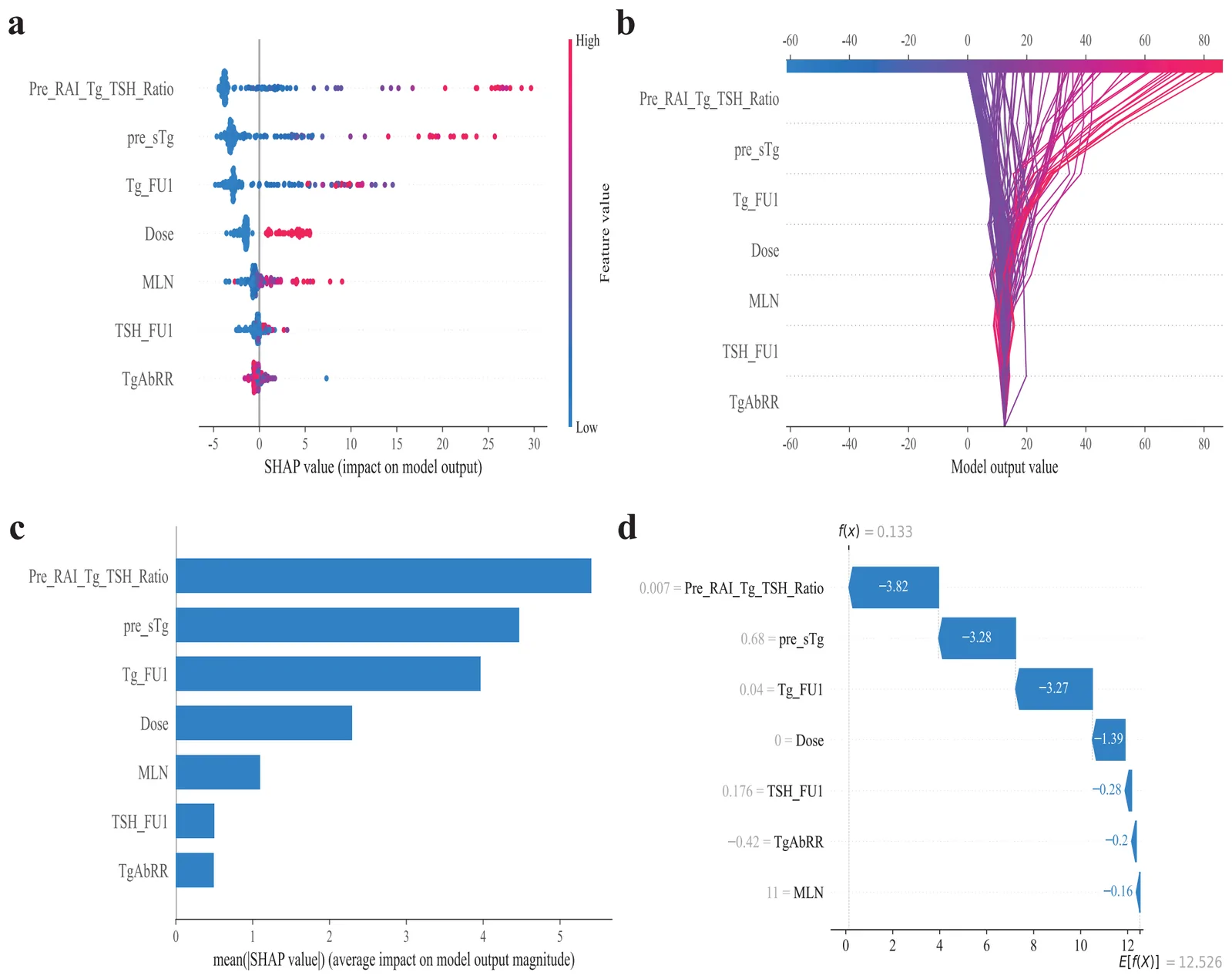
SHAP-based interpretability analysis of the RSF survival prognosis model. **(a)** SHAP beeswarm plot illustrating the distribution and direction of the impact of each feature on the model’s survival output. Each point represents an individual patient, with color representing feature value (blue for low, red for high). **(b)** SHAP decision plot visualizing the cumulative impact of clinical features on the predicted model output for individual patients. **(c)** Global feature importance ranked by mean absolute SHAP values, representing the average magnitude of the contribution of each variable to the prognostic model. **(d)** SHAP waterfall plot demonstrating the individual decision-making process for a representative patient, showing how specific clinical features shift the predicted survival output from the baseline to the final value.

For the RSF model, SHAP analysis was used to assess the relative contributions of pre_sTg, Tg_FU1, Pre_RAI_Tg_TSH_Ratio, MLN, RAI dose, TSH_FU1, and TgAbRR to PRFS prediction. **Figures 7a, c** showed that biochemical variables measured before RAI and during the early post-treatment period, together with nodal disease burden and RAI dose, contributed substantially to the prognostic output. Notably, pre_sTg, MLN, and RAI dose showed prominent contributions in both the classification and survival models. The SHAP decision plot further illustrated how multiple predictor contributions accumulated to produce patient-specific model outputs (**Figure 7b**). At the individual level, the waterfall plot decomposed the RSF prediction for a representative patient into the contribution of each variable relative to the model baseline (**Figure 7d**). The magnitude of contribution from biochemical markers, nodal disease characteristics, and treatment-related factors varied across patients. Thus, PRFS prediction by the RSF model reflected the combined effects of multiple clinical features rather than being driven by a single biomarker.

## 4 Discussion

This study integrated clinical data from 550 patients with DTC who underwent initial RAIT to develop a machine learning-based framework for predicting post-operative therapeutic response and prognosis. In the testing cohort, the ANN model demonstrated superior discriminative ability for identifying early high-risk suboptimal response (BIR/SIR) (AUC = 0.932; 95% CI: 0.888–0.967). Given the 20.5% prevalence of BIR/SIR, the ANN’s NPV of 0.905 indicates that 90.5% of patients classified as having a favorable response at the prespecified 0.50 threshold actually had a favorable outcome in the testing cohort, supporting its potential value for identifying lower-risk patients. Furthermore, for longitudinal prognostic prediction, the RSF model demonstrated favorable time-dependent predictive performance for PRFS (mean AUC = 0.910; 95% CI: 0.867–0.940; C-index = 0.8861), outperforming traditional models such as Cox PH.

Thyroglobulin (Tg) is a glycoprotein secreted by thyroid follicular epithelial cells; in patients who have undergone total thyroidectomy, serum Tg levels primarily reflect residual or recurrent disease. As the main purpose of RAIT is to eliminate residual thyroid tissue and micrometastases, our study observed that patients in the unfavorable response group exhibited significantly higher Pre sTg and follow-up Tg levels. Pre sTg effectively captures the post-operative residual tumor burden and serves as a critical predictor for disease remission, persistence, or recurrence, consistent with previous findings (17–19).

TSH promotes the growth of thyroid cells via the TSH receptor, which remains expressed in most DTC cells. Post-operative TSH-suppressive therapy is therefore essential to both correct hormonal deficiency and inhibit potential tumor growth (20). Our findings indicated that suboptimal TSH suppression (manifested as relatively higher TSH_FU1 levels) at 1–3 months post-RAI was associated with a trend toward unfavorable outcomes, reinforcing the paradigm that maintaining TSH suppression helps mitigate recurrence risk.

We further identified that an elevated Pre sTg/TSH ratio is significantly associated with shorter PRFS. Pathophysiologically, this suggests that under equivalent TSH stimulation, the body secretes more Tg, indicating a larger residual tumor burden and a higher risk of treatment resistance. This finding aligns with recent literature: Zhou et al. (21) identified the Pre sTg/TSH ratio as a reliable diagnostic marker for response to initial RAIT, noting that a ratio >0.127 significantly increases the risk of Non-ER. Similarly, Wen et al. (22) and Li et al. (23) validated Pre sTg/TSH as an independent risk factor for Non-ER in intermediate-to-high-risk DTC, showing that models incorporating this ratio yield higher clinical net benefit compared to those using Pre sTg alone.

Regarding thyroglobulin antibody (TgAb), our study investigated not only its dynamic changes but also the impact of baseline levels on prognosis by incorporating pre-RAI TgAb levels and the TgAb reduction rate (TgAbRR). Rosario et al. (24) demonstrated that patients with a >50% decline in TgAb within one year post-RAIT had a recurrence rate of less than 2%, whereas those with persistently elevated TgAb faced a significantly higher risk. Similarly, we observed that a continuous post-operative decline in TgAb suggests the effective clearance of autoimmune thyroid tissue, indicating a favorable prognosis. In contrast, patients with a poor TgAb reduction rate may harbor occult residual lesions or persistent immune activation, resulting in an unfavorable prognosis. Interestingly, we found that patients with lower pre-RAI TgAb levels often exhibited higher Pre sTg levels and were predicted by our models to have a suboptimal response. This seemingly paradoxical phenomenon can be explained by two factors. First, the proportion of patients with comorbid Hashimoto’s thyroiditis (HT) in the high-risk unfavorable response group was significantly lower than that in the favorable response group (10.13% vs 24.18%, P = 0.007). A meta-analysis by Xu et al. (25) indicated that while HT increases the risk of papillary thyroid carcinoma (PTC), PTC patients with concurrent HT exhibit more favorable clinical characteristics and a better prognosis than those without HT. This implies that lower pre-RAI TgAb levels may reflect the absence of this HT-mediated immune protective effect, rendering the tumor more aggressive and manifesting as a higher tumor burden (high Pre sTg) and poorer treatment response. Second, from an analytical perspective, although we excluded patients with strong TgAb positivity (>115 IU/mL) to avoid severe interference with serum Tg measurements, Tg levels in patients with moderately elevated TgAb may still be partially “masked.” Conversely, for patients with low baseline TgAb, this interference is minimal, allowing Pre sTg to more accurately and sensitively reflect the true residual tumor burden. Therefore, elevated Pre sTg detected under low-TgAb conditions often signifies a heavier true tumor load, ultimately leading our models to predict a suboptimal response.

Notably, the machine learning models identified that higher RAI doses were associated with higher risks of BIR/SIR. This does not imply that “high doses cause poor efficacy,” but rather reflects “indication bias”—a common phenomenon in real-world data. Clinicians typically prescribe higher doses for patients with high-risk features in hopes of achieving better disease control. This “on-demand” clinical logic results in a statistical correlation between dose and poorer prognostic trajectories, confirming that the dose acts as a proxy for baseline disease severity. The high weight assigned to this variable proves that the model accurately captured the clinical decision-making pattern: “high risk, high dose, high attention.”

Regional lymph node involvement is another core determinant of clinical outcomes in patients with DTC. Our study confirmed that the number of MLN and the presence of iodine_LN on post-therapy imaging are critical risk factors for high-risk suboptimal response (BIR/SIR). Using the SHAP framework, we identified that the risk of poor response increases relatively slowly until the number of metastatic lymph nodes reaches approximately 10, beyond which the likelihood of BIR/SIR escalates rapidly. This finding is consistent with the large-scale SEER database study by Sun et al. (26), which demonstrated that the TNnM staging system, which incorporates the number of metastatic lymph nodes (Node number, Nn), provides superior prognostic value compared to the conventional AJCC 8th edition staging system that focuses primarily on anatomical location. We hypothesize that when lymph node metastasis exceeds 10, the disease may have undergone malignant biological progression, harboring significant potential for occult recurrence. As the clinical course advances to the functional evaluation phase after initial RAIT, the iodine-avid status of lymph nodes, as assessed by post-therapy whole-body scintigraphy combined with neck ultrasound, becomes a pivotal prognostic indicator. Notably, all patients in our study underwent standardized total thyroidectomy and regional lymph node dissection. Therefore, the detection of structurally suspicious lymph nodes on post-RAI imaging often signifies stubborn residual micrometastases or early locoregional recurrence, warranting heightened clinical vigilance. Our study revealed a clear risk gradient related to the iodine_LN status. Compared to patients without suspicious lymph nodes, the presence of iodine-avid lymph nodes significantly increases the risk of BIR/SIR, whereas the risk is highest in patients with non-iodine-avid lymph nodes. This phenomenon, characterized by anatomical presence but functional loss of iodine uptake (27), indicates that residual tumor cells have undergone dedifferentiation and potentially acquired resistance to RAIT, signaling a transition toward radioiodine-refractory thyroid cancer and inevitably leading to poorer clinical outcomes.

Although DTC generally carries a more favorable prognosis than many other malignancies, the response to initial RAIT varies significantly among patients. Epidemiological data show that approximately 10% to 30% of patients with DTC experience biochemical or structural incomplete response (BIR/SIR) during long-term follow-up (28), and the 20.5% incidence rate in our study is consistent with these reports. This high-risk population constitutes a group of potential candidates for local recurrence and distant metastasis, necessitating more intensive follow-up and monitoring. Therefore, during the critical therapeutic evaluation window six months post-RAIT, early and precise identification of patients at high risk for BIR/SIR and the prediction of their prognostic trajectories are of great clinical value for timely adjustment of TSH suppression intensity, optimization of imaging follow-up frequency, and even preemptive intervention. To address this core need in RAIT management, we developed a dual-mode machine learning prediction system that achieves comprehensive coverage from static risk identification to dynamic prognostic assessment. First, the risk classification model (ANN) precisely identified the key factors influencing high-risk suboptimal response (BIR/SIR) as Pre sTg, Pre TgAb, MLN, Dose, ETE, iodine_LN, and T-stage. Second, the survival prognosis model (RSF) further revealed the dynamic prognostic factors determining the length of PRFS, including the Pre sTg/TSH ratio, Pre sTg, Tg_FU1, Dose, MLN, TSH_FU1, and TgAbRR. Interestingly, an in-depth analysis using the SHAP interpretability framework revealed that Pre sTg, MLN, and RAI Dose consistently occupied high feature weights in both models. We anticipate that this combination of key predictive variables will more effectively facilitate patient risk and prognostic stratification. Under the core principle of ensuring oncological safety, more precise and multidimensional risk assessments will empower physicians to solidify the “less is more” philosophy regarding treatment for the growing population of low-risk patients. Furthermore, by utilizing intelligent and continuous dynamic risk stratification strategies, clinicians can implement “less monitoring” measures for the vast majority of survivors, thereby improving and optimizing the clinical management of DTC after RAI therapy.

Although this study achieved a favorable predictive performance, several limitations should be acknowledged. First, this study was a single-center retrospective cohort; therefore, future prospective, multicenter, and large-scale external validation is required to further verify the generalizability of our models. Second, the study period extended from August 2021 to June 2025, during which temporal changes in clinical practice may have occurred. Although all patients were retrospectively reassessed using a uniform 2025 ATA classification framework, residual calendar-period effects cannot be entirely excluded. Moreover, given the long natural history of DTC, the median follow-up was only 18.46 months (95% CI, 16.82–20.30 months), indicating that the present PRFS analysis primarily reflects short- to intermediate-term outcomes after RAIT. Longer follow-up is therefore required to evaluate the stability of the models for longer-term prognosis. Finally, our models were constructed based on conventional clinical features. Future research integrating clinical phenotypes, radiomic image features, and multi-omics molecular pathology data through deep learning holds the promise of further elevating the precision medicine of thyroid cancer to new heights.

## 5 Conclusion

This study utilized multidimensional clinical information acquired during the early post-RAI period to develop and internally validate an ANN model for unfavorable-response classification and an RSF model for PRFS prediction. These models constitute an early post-treatment prognostic assessment tool; however, further prospective and external validation is required before clinical implementation.

## Data Availability

The data that support the findings of this study are subject to restrictions imposed by the hospital ethics committee, which does not allow the disclosure of patient-related information. Therefore, the dataset is not publicly available. Reasonable requests for de−identified data may be considered by the corresponding author upon approval from the ethics committee. Requests to access the datasets should be directed to SY, yaoshuzhan@sdfmu.edu.cn.
