# Supplementary material for: Predicting unfavorable response to initial radioactive iodine therapy in differentiated thyroid cancer: an explainable machine learning and survival analysis approach
Source: Front Endocrinol (Lausanne). 2026 Sep 4;17:1943597. doi: 10.3389/fendo.2026.1943597 (PMC13585597; doi:10.3389/fendo.2026.1943597)
Supplement: Supplementary file 1 [file Supplementaryfile1.docx]

Supplementary Material

# Supplementary Methods

**Model Performance Evaluation**

For binary classification, unfavorable response (BIR/SIR) was defined as the positive class and favorable response (ER/IndR) as the negative class. Let TP, TN, FP, and FN denote true positives, true negatives, false positives, and false negatives, respectively, and let N = TP + TN + FP + FN. Threshold-dependent metrics were calculated at the prespecified probability threshold of 0.50 as follows:

Accuracy = (TP + TN) / N

Sensitivity = TP / (TP + FN)	Specificity = TN / (TN + FP)

PPV = TP / (TP + FP)	NPV = TN / (TN + FN)

F1-score = 2 × (PPV × Sensitivity) / (PPV + Sensitivity) = 2TP / (2TP + FP + FN)

Sensitivity represents the proportion of patients with BIR/SIR correctly identified, whereas specificity represents the proportion of patients with a favorable response correctly identified. PPV represents the proportion of patients predicted to have an unfavorable response who actually developed BIR/SIR, whereas NPV represents the proportion of patients predicted to have a favorable response who actually had a favorable outcome. PPV and NPV are prevalence dependent. Accuracy, sensitivity, specificity, PPV, NPV, and F1-score range from 0 to 1, with higher values indicating better performance.

Cohen’s kappa coefficient was calculated as

κ = (Pₒ − Pₑ) / (1 − Pₑ)

where Pₒ is the observed agreement and Pₑ is the agreement expected by chance.

Youden’s J index was calculated as

J = Sensitivity + Specificity − 1

The index ranges from −1 to 1, with higher values indicating a better balance between sensitivity and specificity. In this study, Youden’s J was calculated at the fixed probability threshold of 0.50 and was not used for threshold optimization.

The Brier score was calculated as

Brier score = (1/N) × Σᵢ₌₁ᴺ (pᵢ − yᵢ)²

where pᵢ is the predicted probability for patient i, and yᵢ is the corresponding observed binary outcome. Lower Brier scores indicate better probabilistic accuracy.

The area under the receiver operating characteristic curve (AUC) represents the area under the sensitivity-versus-(1 − specificity) curve across all possible classification thresholds. An AUC of 0.5 indicates no discriminatory ability, whereas an AUC of 1.0 indicates perfect discrimination.

Calibration curves were used to assess agreement between predicted and observed probabilities. Decision Curve Analysis (DCA) was used to evaluate net clinical benefit across a range of threshold probabilities. At a threshold probability pₜ, net benefit was defined as

NB = TP / N − (FP / N) × [pₜ / (1 − pₜ)]

For survival prediction, discrimination was assessed using the concordance index (C-index) and time-dependent AUC. The C-index represents the proportion of comparable patient pairs for which the predicted risk ordering is concordant with the observed event ordering. Time-dependent AUC evaluates discrimination at a specified follow-up horizon. Time-dependent calibration curves were used to assess agreement between predicted and observed event probabilities at 9, 12, 18, and 24 months, whereas time-dependent DCA evaluated net clinical benefit across threshold probabilities at the corresponding time points.

# Supplementary Tables

# Table S1.Baseline clinical characteristics and persistence/recurrence-free of survival patients with differentiated thyroid carcinoma.

| **Characteristics** | **Total (n = 550)** | **test (n = 165)** | **train (n = 385)** | **χ2/t/Z** | ***P*** |
| --- | --- | --- | --- | --- | --- |
| BMI (kg/m²), mean (SD) | 25.34 ± 3.92 | 25.32 ± 4.09 | 25.34 ± 3.85 | t=-0.07 | 0.946 |
| Age (years), median (IQR) | 42.00 (34.00, 51.75) | 44.00 (35.00, 53.00) | 41.00 (33.00, 51.00) | Z=-1.92 | 0.054 |
| Glu (mmol/L), median (IQR) | 4.86 (4.42, 5.38) | 4.85 (4.36, 5.49) | 4.87 (4.45, 5.38) | Z=-0.20 | 0.845 |
| TG (mmol/L), median (IQR) | 0.50 (0.46, 1.14) | 0.50 (0.46, 1.16) | 0.53 (0.46, 1.13) | Z=-0.48 | 0.634 |
| TC (mmol/L), median (IQR) | 2.79 (2.39, 4.76) | 2.72 (2.39, 4.65) | 3.02 (2.39, 4.76) | Z=-0.58 | 0.564 |
| HDL (mmol/L), median (IQR) | 0.96 (0.68, 1.37) | 0.90 (0.68, 1.38) | 0.96 (0.68, 1.34) | Z=-0.24 | 0.808 |
| LDL (mmol/L), median (IQR) | 3.39 (2.35, 5.09) | 3.33 (2.34, 5.09) | 3.49 (2.37, 5.26) | Z=-1.06 | 0.290 |
| TyG, median (IQR) | 7.48 (7.34, 8.41) | 7.42 (7.34, 8.31) | 7.55 (7.34, 8.41) | Z=-0.74 | 0.459 |
| Pathological tumor size (cm), median (IQR) | 1.20 (0.80, 1.80) | 1.20 (0.80, 2.20) | 1.20 (0.80, 1.80) | Z=-0.81 | 0.418 |
| Metastatic lymph nodes, median (IQR) | 5.00 (3.00, 10.00) | 5.00 (3.00, 10.00) | 5.00 (3.00, 10.00) | Z=-0.69 | 0.488 |
| ELN, median [IQR] | 18.00 (8.00, 30.00) | 17.00 (8.00, 31.00) | 18.00 (8.00, 30.00) | Z=-0.09 | 0.927 |
| LNR, median [IQR] | 0.36 (0.21, 0.57) | 0.36 (0.20, 0.56) | 0.36 (0.23, 0.58) | Z=-0.50 | 0.615 |
| Surgery‑to‑RAI interval (months), median (IQR) | 3.55 (2.53, 4.80) | 3.68 (2.53, 5.52) | 3.42 (2.53, 4.73) | Z=-1.22 | 0.221 |
| Pre‑RAI TSH (μIU/mL), median (IQR) | 89.10 (65.53, 100.00) | 83.29 (67.15, 100.00) | 92.21 (65.22, 100.00) | Z=-0.29 | 0.771 |
| Pre‑RAI sTg (ng/mL), median (IQR) | 3.92 (0.81, 12.47) | 4.00 (0.80, 12.20) | 3.84 (0.85, 12.60) | Z=-0.35 | 0.727 |
| Pre‑RAI TgAb (IU/mL), median (IQR) | 15.30 (13.60, 18.85) | 15.50 (13.90, 18.70) | 15.30 (13.50, 19.00) | Z=-1.05 | 0.295 |
| Pre‑RAI Tg/TSH ratio, median (IQR) | 0.05 (0.01, 0.16) | 0.05 (0.01, 0.20) | 0.05 (0.01, 0.16) | Z=-0.32 | 0.748 |
| RAI FU1 interval (months), median (IQR) | 1.22 (1.12, 1.45) | 1.22 (1.12, 1.45) | 1.22 (1.12, 1.45) | Z=-0.12 | 0.901 |
| TSH FU1 (μIU/mL), median (IQR) | 0.32 (0.10, 1.57) | 0.24 (0.10, 1.15) | 0.35 (0.10, 1.81) | Z=-1.16 | 0.245 |
| Tg FU1 (ng/mL), median (IQR) | 0.25 (0.04, 1.30) | 0.28 (0.04, 1.38) | 0.24 (0.04, 1.20) | Z=-0.44 | 0.663 |
| TgAb FU1 (IU/mL), median (IQR) | 15.80 (13.60, 19.88) | 16.30 (13.90, 20.20) | 15.60 (13.60, 19.40) | Z=-1.10 | 0.270 |
| TgRR (median [IQR]) | 0.90 (0.73, 0.96) | 0.91 (0.77, 0.96) | 0.89 (0.73, 0.96) | Z=-0.69 | 0.493 |
| TgAbRR (median [IQR]) | -0.01 (-0.12, 0.10) | -0.01 (-0.14, 0.13) | -0.02 (-0.12, 0.10) | Z=-0.27 | 0.785 |
| PRFS (months), median (IQR) | 15.21 (8.97, 23.92) | 13.86 (8.94, 21.72) | 15.47 (8.97, 24.44) | Z=-1.20 | 0.231 |
| Group(%) |  |  |  | χ²=0.00 | 0.982 |
| Low Risk (ER/IndR) | 437 (79.45) | 131 (79.39) | 306 (79.48) |  |  |
| High Risk (BIR/SIR) | 113 (20.55) | 34 (20.61) | 79 (20.52) |  |  |
| Sex(%) |  |  |  | χ²=0.09 | 0.764 |
| Female | 375 (68.18) | 114 (69.09) | 261 (67.79) |  |  |
| Male | 175 (31.82) | 51 (30.91) | 124 (32.21) |  |  |
| HBP(%) |  |  |  | χ²=0.20 | 0.655 |
| NO | 469 (85.27) | 139 (84.24) | 330 (85.71) |  |  |
| YES | 81 (14.73) | 26 (15.76) | 55 (14.29) |  |  |
| Diabetes(%) |  |  |  | χ²=1.53 | 0.216 |
| NO | 516 (93.82) | 158 (95.76) | 358 (92.99) |  |  |
| YES | 34 (6.18) | 7 (4.24) | 27 (7.01) |  |  |
| Hashimoto(%) |  |  |  | χ²=0.26 | 0.614 |
| NO | 436 (79.27) | 133 (80.61) | 303 (78.70) |  |  |
| YES | 114 (20.73) | 32 (19.39) | 82 (21.30) |  |  |
| Dose(%) |  |  |  | χ²=0.32 | 0.569 |
| 100mCi | 363 (66.00) | 106 (64.24) | 257 (66.75) |  |  |
| 150mCi | 187 (34.00) | 59 (35.76) | 128 (33.25) |  |  |
| Type(%) |  |  |  | - | 0.181 |
| Low-risk PTMC | 101 (18.36) | 30 (18.18) | 71 (18.44) |  |  |
| Classic PTC (>1 cm) | 415 (75.45) | 127 (76.97) | 288 (74.81) |  |  |
| Follicular variant PTC (>1 cm) | 19 (3.45) | 2 (1.21) | 17 (4.42) |  |  |
| High-risk PTC (any size) | 8 (1.45) | 2 (1.21) | 6 (1.56) |  |  |
| FTC | 7 (1.27) | 4 (2.42) | 3 (0.78) |  |  |
| Multifocality(%) |  |  |  | χ²=0.96 | 0.326 |
| NO | 173 (31.45) | 47 (28.48) | 126 (32.73) |  |  |
| YES | 377 (68.55) | 118 (71.52) | 259 (67.27) |  |  |
| Bilaterality(%) |  |  |  | χ²=3.32 | 0.069 |
| NO | 239 (43.45) | 62 (37.58) | 177 (45.97) |  |  |
| YES | 311 (56.55) | 103 (62.42) | 208 (54.03) |  |  |
| ETE(%) |  |  |  | χ²=1.28 | 0.528 |
| NO | 254 (46.18) | 79 (47.88) | 175 (45.45) |  |  |
| Invasion into capsule | 225 (40.91) | 62 (37.58) | 163 (42.34) |  |  |
| Invasion through capsule | 71 (12.91) | 24 (14.55) | 47 (12.21) |  |  |
| T(%) |  |  |  | χ²=8.42 | 0.038 |
| T1a | 234 (42.55) | 67 (40.61) | 167 (43.38) |  |  |
| T1b | 215 (39.09) | 56 (33.94) | 159 (41.30) |  |  |
| T2 | 80 (14.55) | 34 (20.61) | 46 (11.95) |  |  |
| T3a | 21 (3.82) | 8 (4.85) | 13 (3.38) |  |  |
| N(%) |  |  |  | χ²=0.28 | 0.868 |
| N0 | 17 (3.09) | 6 (3.64) | 11 (2.86) |  |  |
| N1a | 184 (33.45) | 56 (33.94) | 128 (33.25) |  |  |
| N1b | 349 (63.45) | 103 (62.42) | 246 (63.90) |  |  |
| M(%) |  |  |  | - | 1.000 |
| M0 | 547 (99.45) | 164 (99.39) | 383 (99.48) |  |  |
| M1 | 3 (0.55) | 1 (0.61) | 2 (0.52) |  |  |
| AJCC(%) |  |  |  | - | 0.280 |
| I | 460 (83.64) | 132 (80.00) | 328 (85.19) |  |  |
| II | 84 (15.27) | 32 (19.39) | 52 (13.51) |  |  |
| III | 5 (0.91) | 1 (0.61) | 4 (1.04) |  |  |
| IVA | 1 (0.18) | 0 (0.00) | 1 (0.26) |  |  |
| Iodine Ln(%) |  |  |  | - | 0.167 |
| No suspicious lymph nodes | 380 (69.09) | 108 (65.45) | 272 (70.65) |  |  |
| Iodine‑avid lymph nodes | 167 (30.36) | 55 (33.33) | 112 (29.09) |  |  |
| Non‑iodine‑avid lymph nodes | 3 (0.55) | 2 (1.21) | 1 (0.26) |  |  |
| ATA Ris(%) |  |  |  | - | 0.026 |
| Low risk | 2 (0.36) | 2 (1.21) | 0 (0.00) |  |  |
| Low-intermediate risk | 232 (42.18) | 58 (35.15) | 174 (45.19) |  |  |
| Intermediate-high risk | 301 (54.73) | 101 (61.21) | 200 (51.95) |  |  |
| High risk | 15 (2.73) | 4 (2.42) | 11 (2.86) |  |  |

# Table S2.Baseline Clinical Characteristics of the Training Cohort in Patients with Differentiated Thyroid Cancer

| **Characteristics** | **Total (n = 385)** | **Low Risk (ER/IndR)(n = 306)** | **High Risk (BIR/SIR) (n = 79)** | **χ2/t/Z** | ***P*** |
| --- | --- | --- | --- | --- | --- |
| BMI (kg/m²), mean (SD) | 25.34 ± 3.85 | 25.20 ± 3.93 | 25.92 ± 3.50 | t=-1.49 | 0.138 |
| Age (years), median (IQR) | 41.00 (33.00, 51.00) | 41.00 (34.00, 50.75) | 41.00 (32.50, 50.50) | Z=-0.71 | 0.478 |
| Glu (mmol/L), median (IQR) | 4.87 (4.45, 5.38) | 4.87 (4.44, 5.38) | 4.83 (4.46, 5.36) | Z=-0.22 | 0.825 |
| TG (mmol/L), median (IQR) | 0.53 (0.46, 1.13) | 0.51 (0.46, 1.10) | 0.64 (0.48, 1.25) | Z=-1.52 | 0.129 |
| TC (mmol/L), median (IQR) | 3.02 (2.39, 4.76) | 2.79 (2.39, 4.67) | 3.52 (2.61, 4.95) | Z=-1.14 | 0.254 |
| HDL (mmol/L), median (IQR) | 0.96 (0.68, 1.34) | 0.81 (0.68, 1.33) | 1.07 (0.70, 1.39) | Z=-2.03 | 0.042 |
| LDL (mmol/L), median (IQR) | 3.49 (2.37, 5.26) | 3.29 (2.25, 5.09) | 3.96 (2.89, 5.26) | Z=-2.67 | 0.008 |
| TyG, median (IQR) | 7.55 (7.34, 8.41) | 7.48 (7.34, 8.41) | 7.68 (7.34, 8.54) | Z=-0.94 | 0.346 |
| Pathological tumor size (cm), median (IQR) | 1.20 (0.80, 1.80) | 1.10 (0.80, 1.50) | 1.50 (1.00, 2.90) | Z=-5.11 | <.001 |
| Metastatic lymph nodes, median (IQR) | 5.00 (3.00, 10.00) | 5.00 (3.00, 9.00) | 11.00 (5.00, 16.00) | Z=-5.44 | <.001 |
| ELN, median [IQR] | 18.00 (8.00, 30.00) | 17.00 (8.00, 28.00) | 22.00 (11.50, 36.00) | Z=-2.42 | 0.015 |
| LNR, median [IQR] | 0.36 (0.23, 0.58) | 0.33 (0.21, 0.54) | 0.48 (0.33, 0.67) | Z=-3.33 | <.001 |
| Surgery‑to‑RAI interval (months), median (IQR) | 3.42 (2.53, 4.73) | 3.55 (2.53, 4.80) | 3.15 (2.46, 4.21) | Z=-1.48 | 0.138 |
| Pre‑RAI TSH (μIU/mL), median (IQR) | 92.21 (65.22, 100.00) | 93.33 (64.73, 100.00) | 86.70 (65.32, 100.00) | Z=-0.16 | 0.870 |
| Pre‑RAI sTg (ng/mL), median (IQR) | 3.84 (0.85, 12.60) | 2.42 (0.46, 6.74) | 39.90 (12.62, 133.65) | Z=-11.14 | <.001 |
| Pre‑RAI TgAb (IU/mL), median (IQR) | 15.30 (13.50, 19.00) | 15.80 (13.60, 22.28) | 14.10 (13.00, 15.90) | Z=-4.01 | <.001 |
| Pre‑RAI Tg/TSH ratio, median (IQR) | 0.05 (0.01, 0.16) | 0.03 (0.01, 0.08) | 0.49 (0.15, 1.73) | Z=-11.16 | <.001 |
| RAI FU1 interval (months), median (IQR) | 1.22 (1.12, 1.45) | 1.22 (1.12, 1.45) | 1.22 (1.12, 1.40) | Z=-0.48 | 0.629 |
| TSH FU1 (μIU/mL), median (IQR) | 0.35 (0.10, 1.81) | 0.36 (0.10, 1.96) | 0.30 (0.08, 1.39) | Z=-1.39 | 0.164 |
| Tg FU1 (ng/mL), median (IQR) | 0.24 (0.04, 1.20) | 0.12 (0.04, 0.53) | 3.71 (1.61, 9.57) | Z=-11.17 | <.001 |
| TgAb FU1 (IU/mL), median (IQR) | 15.60 (13.60, 19.40) | 16.10 (13.60, 22.07) | 14.40 (13.45, 16.11) | Z=-3.45 | <.001 |
| TgRR (median [IQR]) | 0.89 (0.73, 0.96) | 0.89 (0.70, 0.96) | 0.91 (0.80, 0.95) | Z=-1.18 | 0.237 |
| TgAbRR (median [IQR]) | -0.02 (-0.12, 0.10) | -0.01 (-0.12, 0.11) | -0.03 (-0.12, 0.07) | Z=-0.79 | 0.432 |
| PRFS (months), median (IQR) | 15.47 (8.97, 24.44) | 17.79 (10.00, 26.36) | 9.66 (7.62, 13.78) | Z=-6.08 | <.001 |
| Sex(%) |  |  |  | χ²=3.13 | 0.077 |
| Female | 261 (67.79) | 214 (69.93) | 47 (59.49) |  |  |
| Male | 124 (32.21) | 92 (30.07) | 32 (40.51) |  |  |
| HBP(%) |  |  |  | χ²=0.22 | 0.643 |
| NO | 330 (85.71) | 261 (85.29) | 69 (87.34) |  |  |
| YES | 55 (14.29) | 45 (14.71) | 10 (12.66) |  |  |
| Diabetes(%) |  |  |  | χ²=0.58 | 0.447 |
| NO | 358 (92.99) | 283 (92.48) | 75 (94.94) |  |  |
| YES | 27 (7.01) | 23 (7.52) | 4 (5.06) |  |  |
| Hashimoto(%) |  |  |  | χ²=7.40 | 0.007 |
| NO | 303 (78.70) | 232 (75.82) | 71 (89.87) |  |  |
| YES | 82 (21.30) | 74 (24.18) | 8 (10.13) |  |  |
| Dose(%) |  |  |  | χ²=96.84 | <.001 |
| 100mCi | 257 (66.75) | 241 (78.76) | 16 (20.25) |  |  |
| 150mCi | 128 (33.25) | 65 (21.24) | 63 (79.75) |  |  |
| Type(%) |  |  |  | - | 0.145 |
| Low-risk PTMC | 71 (18.44) | 63 (20.59) | 8 (10.13) |  |  |
| Classic PTC (>1 cm) | 288 (74.81) | 224 (73.20) | 64 (81.01) |  |  |
| Follicular variant PTC (>1 cm) | 17 (4.42) | 12 (3.92) | 5 (6.33) |  |  |
| High-risk PTC (any size) | 6 (1.56) | 5 (1.63) | 1 (1.27) |  |  |
| FTC | 3 (0.78) | 2 (0.65) | 1 (1.27) |  |  |
| Multifocality(%) |  |  |  | χ²=0.25 | 0.618 |
| NO | 126 (32.73) | 102 (33.33) | 24 (30.38) |  |  |
| YES | 259 (67.27) | 204 (66.67) | 55 (69.62) |  |  |
| Bilaterality(%) |  |  |  | χ²=0.71 | 0.401 |
| NO | 177 (45.97) | 144 (47.06) | 33 (41.77) |  |  |
| YES | 208 (54.03) | 162 (52.94) | 46 (58.23) |  |  |
| ETE(%) |  |  |  | χ²=40.61 | <.001 |
| NO | 175 (45.45) | 151 (49.35) | 24 (30.38) |  |  |
| Invasion into capsule | 163 (42.34) | 134 (43.79) | 29 (36.71) |  |  |
| Invasion through capsule | 47 (12.21) | 21 (6.86) | 26 (32.91) |  |  |
| T(%) |  |  |  | χ²=40.75 | <.001 |
| T1a | 167 (43.38) | 146 (47.71) | 21 (26.58) |  |  |
| T1b | 159 (41.30) | 130 (42.48) | 29 (36.71) |  |  |
| T2 | 46 (11.95) | 26 (8.50) | 20 (25.32) |  |  |
| T3a | 13 (3.38) | 4 (1.31) | 9 (11.39) |  |  |
| N(%) |  |  |  | χ²=12.64 | 0.002 |
| N0 | 11 (2.86) | 10 (3.27) | 1 (1.27) |  |  |
| N1a | 128 (33.25) | 114 (37.25) | 14 (17.72) |  |  |
| N1b | 246 (63.90) | 182 (59.48) | 64 (81.01) |  |  |
| M(%) |  |  |  | - | 0.042 |
| M0 | 383 (99.48) | 306 (100.00) | 77 (97.47) |  |  |
| M1 | 2 (0.52) | 0 (0.00) | 2 (2.53) |  |  |
| AJCC(%) |  |  |  | - | 0.019 |
| I | 328 (85.19) | 263 (85.95) | 65 (82.28) |  |  |
| II | 52 (13.51) | 42 (13.73) | 10 (12.66) |  |  |
| III | 4 (1.04) | 1 (0.33) | 3 (3.80) |  |  |
| IVA | 1 (0.26) | 0 (0.00) | 1 (1.27) |  |  |
| Iodine Ln(%) |  |  |  | - | <.001 |
| No suspicious lymph nodes | 272 (70.65) | 233 (76.14) | 39 (49.37) |  |  |
| Iodine‑avid lymph nodes | 112 (29.09) | 72 (23.53) | 40 (50.63) |  |  |
| Non‑iodine‑avid lymph nodes | 1 (0.26) | 1 (0.33) | 0 (0.00) |  |  |
| ATA Ris(%) |  |  |  | χ²=34.69 | <.001 |
| Low-intermediate risk | 174 (45.19) | 144 (47.06) | 30 (37.97) |  |  |
| Intermediate-high risk | 200 (51.95) | 161 (52.61) | 39 (49.37) |  |  |
| High risk | 11 (2.86) | 1 (0.33) | 10 (12.66) |  |  |

# Table S3. Optimal hyperparameter configurations of the classification models.

| **Model** | **Hyperparameter** | **Search space / setting** | **Final setting** |
| --- | --- | --- | --- |
| DT | max_depth | 3, 4, 5, 6, 7 | 3 |
|  | min_samples_split | 10, 20, 30, 50 | 10 |
|  | min_samples_leaf | 5, 10, 15, 20 | 20 |
|  | max_features | sqrt, log2, None | None |
|  | ccp_alpha | 0, 0.005, 0.01, 0.02 | 0 |
|  | class_weight | — | balanced |
|  | random_state | — | 123 |
| RF | n_estimators | 100, 300, 500 | 500 |
|  | max_depth | 3, 5, 7, 10 | 3 |
|  | min_samples_split | 10, 20, 30 | 10 |
|  | min_samples_leaf | 5, 10, 15 | 5 |
|  | max_features | sqrt, log2 | sqrt |
|  | class_weight | — | balanced_subsample |
|  | random_state | — | 211 |
| LightGBM | learning_rate | 0.01, 0.05 | 0.05 |
|  | n_estimators | 100, 200 | 100 |
|  | max_depth | 3, 4, 5 | 3 |
|  | num_leaves | 7, 15, 31 | 15 |
|  | min_child_samples | 10, 20, 30 | 10 |
|  | subsample | 0.7, 0.8 | 0.7 |
|  | colsample_bytree | 0.7, 0.8 | 0.8 |
|  | reg_lambda | 0.1, 1.0 | 1 |
|  | class_weight | — | balanced |
|  | random_state | — | 123 |
| ANN (MLP) | hidden_layer_sizes | (25), (50), (100), (10,10), (50,50), (100,100), (50,50,50) | (10,10) |
|  | activation | ReLU, tanh, logistic | logistic |
|  | solver | — | Adam |
|  | learning_rate_init | — | 0.001 |
|  | alpha | — | 0.0001 |
|  | batch_size | — | auto |
|  | max_iter | — | 500 |
|  | random_state | — | 123 |

**Note:** DT, decision tree; RF, random forest; LightGBM, Light Gradient Boosting Machine; ANN, artificial neural network; MLP, multi-layer perceptron. “—” indicates that the parameter was not included in the grid-search space.

# Table S4. Clinical characteristics and observed outcomes of the five testing-cohort patients illustrated in Figures 5d1 and 5d2

| **Case** | **Study ID** | **pre_sTg** | **Tg_FU1** | **Pre_RAI_Tg_TSH_Ratio** | **MLN** | **RAI dose (mCi)** | **TSH_FU1** | **TgAbRR** | **Observed PRFS status** | **Event/censoring time (months)** |
| --- | --- | --- | --- | --- | --- | --- | --- | --- | --- | --- |
| **Case 1** | 214 | 0.68 | 0.04 | 0.007 | 11 | 100 | 0.1764 | −0.42 | Censored | 12.48 |
| **Case 2** | 305 | 51.71 | 1.4 | 0.517 | 17 | 150 | 3.3468 | 0.13 | Event | 8.97 |
| **Case 3** | 121 | 20.9 | 3.15 | 0.209 | 8 | 150 | 0.1733 | −0.01 | Censored | 10.41 |
| **Case 4** | 143 | 25.6 | 1.03 | 0.336 | 16 | 150 | 0.0188 | −0.16 | Event | 13.21 |
| **Case 5** | 311 | 9.47 | 2.44 | 0.127 | 7 | 150 | 7.1354 | −0.19 | Censored | 6.18 |

**Note**: The five cases correspond, in the same order, to the five randomly selected testing-cohort patients displayed in Figures 5d1 and 5d2. PRFS was defined as the interval from initial radioactive iodine therapy to the first subsequent documentation of biochemical incomplete response (BIR) or structural incomplete response (SIR). “Event” indicates documented BIR/SIR during follow-up, whereas “Censored” indicates that no PRFS event was documented before the last available follow-up; for censored patients, the reported time therefore represents the time to the last follow-up.
